# Crystal structures and hydrogen bonding in the proton-transfer salts of nicotine with 3,5-di­nitro­salicylic acid and 5-sulfosalicylic acid

**DOI:** 10.1107/S1600536814023253

**Published:** 2014-10-29

**Authors:** Graham Smith, Urs D. Wermuth

**Affiliations:** a Science and Engineering Faculty, Queensland University of Technology, GPO Box 2434, Brisbane, Queensland 4001, Australia

**Keywords:** crystal structure, nicotine, proton-transfer salts, 3,5-di­nitro­salicylic acid, 5-sulfosalicylic acid, hydrogen-bonding, π–π inter­actions

## Abstract

The structures of the 1:1 anhydrous salts of nicotine (NIC) with 3,5-di­nitro­salicylic acid (DNSA) and 5-sulfosalicylic acid (5-SSA), namely (1*R*,2*S*)-1-methyl-2-(pyridin-3-yl)-1*H*-pyrrolidin-1-ium 2-carb­oxy-4,6-di­nitro­phenolate, C_10_H_15_N_2_^+^·C_7_H_3_N_2_O_7_^−^, (I), and (1*R*,2*S*)-1-methyl-2-(pyridin-3-yl)-1*H*-pyrrolidin-1-ium 3-carb­oxy-4-hy­droxy­benzene­sulfonate, C_10_H_15_N_2_^+^·C_7_H_5_O_6_S^−^, (II), are reported. The asymmetric units of both (I) and (II) comprise two independent nicotinium cations (*C* and *D*) and either two DNSA or two 5-SSA anions (*A* and *B*), respectively. One of the DNSA anions shows a 25% rotational disorder in the benzene ring system. In the crystal of (I), inter-unit pyrrolidinium N—H⋯N_pyridine_ hydrogen bonds generate zigzag NIC cation chains which extend along *a*, while the DNSA anions are not involved in any formal inter-species hydrogen bonding but instead form π–π-associated stacks which are parallel to the NIC cation chains along *a* [ring-centroid separation = 3.857 (2) Å]. Weak C—H⋯O inter­actions between chain substructures give an overall three-dimensional structure. In the crystal of (II), *A* and *B* anions form independent zigzag chains with *C* and *D* cations, respectively, through carb­oxy­lic acid O—H⋯N_pyridine_ hydrogen bonds. These chains, which extend along *b*, are pseudocentrosymmetrically related and give π–π inter­actions between the benzene rings of anions *A* and *B* and the pyridine rings of the NIC cations *C* and *D*, respectively [ring centroid separations = 3.6422 (19) and 3.7117 (19) Å]. Also present are weak C—H⋯O hydrogen-bonding inter­actions between the chains, giving an overall three-dimensional structure.

## Abstract 2

The crystal structures of the 1:1 salts of nicotine with 3,5-di­nitro­salicylic acid and with 5-sulfosalicylic acid both show polymeric hydrogen-bonded and π–π-bonded structures but these differ in that in the first example, cations and anions form separate cation chains or anion columns which are unassociated through formal hydrogen bonds while in the second, hydrogen-bonded cation–anion chains are found.

## Chemical context   

Nicotine [3-(2*S*-1-methyl­pyrrolidin-2-yl)pyridine] is well known as a toxic liquid alkaloid which is found in the leaves of the tobacco plants *Nicotiana tabacum* and *N. rustica* (Rodgman & Parfetti, 2009 ▶). Because of these properties, nicotine and its compounds have been of commercial inter­est and have been used in the past as insecticides and as veterinary ectoparasiticides (usually as the sulfate) (Ujváry, 1999 ▶), as well as in limited medical applications as the bitartrate (Eudermol) for the treatment of smoking-withdrawal syndrome (Enzell *et al.*, 1977 ▶). However, its veterinary use is restricted due to its toxicity with even topical applications, resulting in the total ban on its use in the USA early in 2014.

As a Lewis base, nicotine is potentially capable of forming both monocationic and dicationic species (p*K*_a1_ = 3.10 and p*K*_a2_ = 8.01) and the sulfate, di­hydro­chloride, bitartrate and bipicrate salts have been reported (O’Neil, 2001 ▶). However, the only example of a simple dicationic salt in the crystallographic literature is the di­hydro­iodide (Koo & Kim, 1965 ▶). Some metal complexes with the dication as a counter-ion are known, *e.g.* tetra­chlorido­copper(II) nicotinate (Choi *et al.*, 2002 ▶). More commonly, monocationic salt structures are reported, *e.g.* the iodide (Barlow *et al.*, 1986 ▶), the picrate (Arnaud *et al.*, 2007 ▶) and the salicylate (Kim & Jeffrey, 1971 ▶).

3,5-Di­nitro­salicylic acid (DNSA) (p*K*_a_ = 2.18) and 3-carb­oxy-4-hy­droxy­benzene­sulfonic acid (5-sulfosalicylic acid: 5-SSA) (p*K*_a_ < 1) are capable of forming salts with most Lewis bases and have been used for the formation of crystalline salts suitable for X-ray analysis, *e.g.* with 5-SSA (Baskar Raj *et al.*, 2003 ▶; Smith *et al.*, 2006 ▶) and with DNSA, where the majority of the salts formed are phenolates rather than carboxyl­ates (Smith *et al.*, 2007 ▶). The title salts C_10_H_15_N_2_^+^ C_7_H_3_N_2_O_7_^−^, (I) and C_10_H_15_N_2_^+^ C_7_H_5_O_6_S^−^, (II) were prepared from the reaction of nicotine (NIC) with DNSA and with 5-SSA, respectively, and the structures are reported herein.
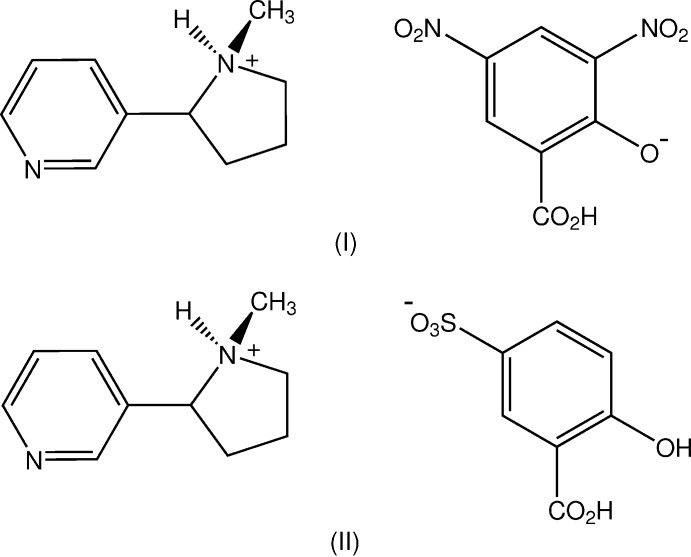


## Structural commentary   

**Figure 1:**
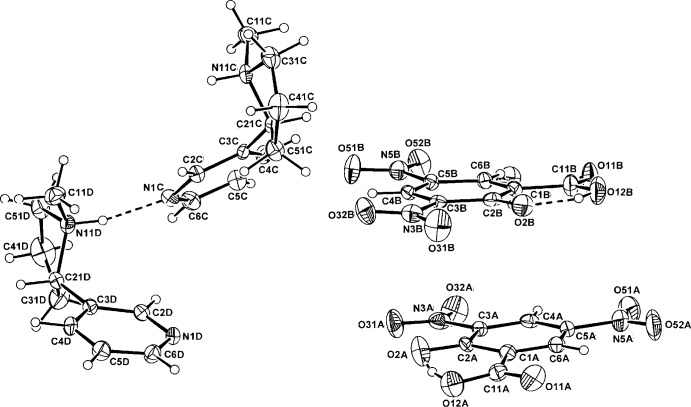
The mol­ecular conformation and atom labelling for the two NIC cations (*C* and *D*) and the two DNSA anions (*A* and *B*) in the asymmetric unit of (I), with displacement ellipsoids drawn at the 40% probability level. Inter-species hydrogen bonds are shown as dashed lines (see Table 1 ▶).

In both the nicotinium salts of DNSA (I) and 5-SSA (II), proton-transfer to the pyrrolidine N-atom of nicotine has occurred as expected, generating an N11(*R*) chiral centre relative to the known C21(*S*) centre. Also, in both (I) and (II) (Figs. 1 ▶ and 2 ▶), the asymmetric units comprise two independent NIC^+^ cations (*C* and *D*) and either, for (I), two DNSA phenolate monoanions or two 5-SSA carboxyl­ate monoanions (*A* and *B*) (Figs. 1 ▶, 2 ▶). With (II), the two independent anion and cation pairs are pseudo-centrosymmetrically related but the presence of the inversion centre is obviated by the fact that both of the NIC cations have the same N11(*R*), C21(*S*) absolute configuration.

In (I), the nicotinium *C* and *D* cations are conformationally similar but in (II), they are different. However, in both, the pyrrolidinium plane is significantly rotated with respect to that of the benzene ring [the torsion angles C2*C/D*—C3*C/D*—C21*C/D*—N11*C/D are* −71.9 (4) (*C*) and −68.8 (4)° (*D*) in (I) and −45.7 (4) (*C*) and 125.7 (3)° (*D*) in (II)]. This conformation with the two rings *anti­planar* is usual for cationic nicotine structures, *e.g.* Arnaud *et al.* (2007 ▶). The substituent carboxyl and nitro groups of the DNSA anions in (I) are essentially coplanar with the benzene ring, with the maximum deviation among the three defining torsion angles for each anion (C2*A/B*—C1*A/B*—C11*A/B*—O2*A/B*, C2*A/B* —C3*A/B*—N3*A/B*—O32*A/B* and C4*A/B*—C5*A/B*—N5*A/B*—O52*A/B*) being for the C3*B* nitro group [173.7 (3)°]. In the *B* anion, there is 25% rotational disorder about the C1⋯C4 ring vector, which generates a second phenolic O-component (O21*B*). This phenomenon has precedence in DNSA salt structures, *e.g.* with the nicotinamide salt (Koman *et al.*, 2003 ▶; 24% disorder). The C3 nitro group is most often associated with deviation from planarity in the DNSA phenolate salts (Smith *et al.*, 2007 ▶) and is the more inter­active and sterically crowded group. In the case of (I), the uncommon planarity is probably associated with the presence of anion π-bonding associations.

With the 5-SSA anions, the carb­oxy­lic acid group is essentially coplanar with the benzene ring, which is expected in this salicylic acid species, invariably having the short intra­molecular carb­oxy­lic acid O—H⋯O_phenol_ hydrogen bond (Table 2 ▶) (Smith *et al.*, 2006 ▶). This inter­action is also present in the phenolate anion in (I) in which the carb­oxy­lic acid H-atom is *anti*-related (Table 1 ▶).

## Supra­molecular features   

In the supra­molecular structure of (I), the two independent NIC cations *C* and *D* inter­act through N1*C* —H⋯N11*D* ^i^ and N1*D* —H⋯N11*C* hydrogen bonds (Table 1 ▶), giving zigzag chains extending along *a* (Fig. 3 ▶). With the DNSA anions, there are no formal hydrogen-bonding inter­actions either between *A* and *B* anions or with the NIC chain structures. Instead, these anions form π–π-bonded stacks which are parallel to the NIC^+^ chains down *a* [ring-centroid separation = 3.857 (2) Å]. The presence of π–π stacking is unusual in DNSA cation structures. In the crystal, there are a number of inter­molecular C*C/D*—H⋯O*A/B* hydrogen-bonding inter­actions, which give an overall three-dimensional structure.

**Figure 2:**
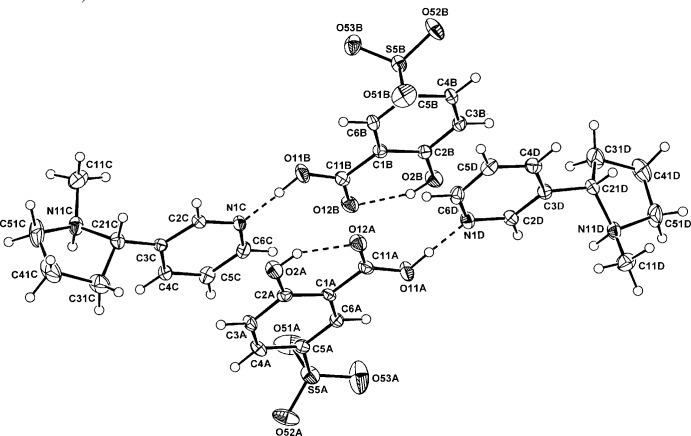
The mol­ecular conformation and atom labelling for the two NIC cations (*C* and *D*) and the two 5-SSA anions (*A* and *B*) in the asymmetric unit of (II), with displacement ellipsoids drawn at the 40% probability level. Inter-species hydrogen bonds are shown as dashed lines (see Table 2 ▶).

**Figure 3:**
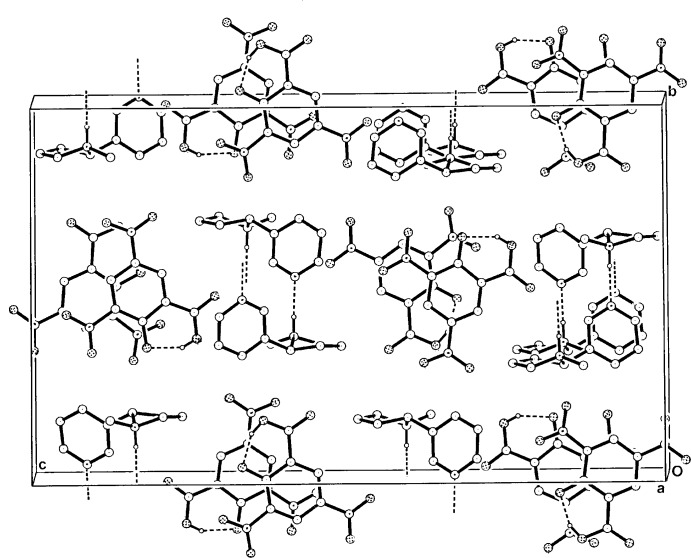
The alternating hydrogen-bonded *C–D* cationic columns and π-bonded *A–B* anion stacks in the structure of (I), viewed along the stacks in the unit cell.

**Figure 4:**
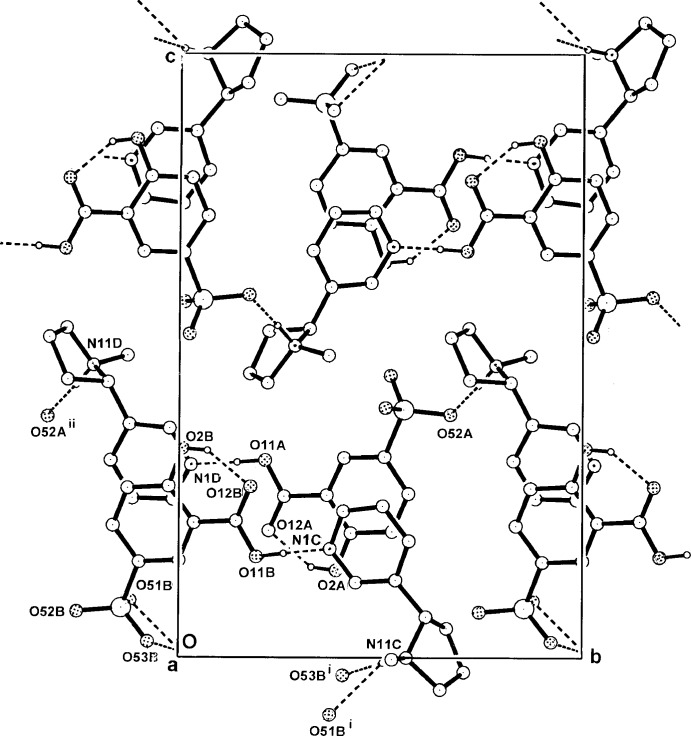
The hydrogen-bonded *A–C* and *B–D* chain structures in (II), extending along *b*. Non-associative H atoms have been omitted. For symmetry codes, see Table 2 ▶.

In the crystal of (II), the independent *A* and *B* 5-SSA anions form carb­oxy­lic acid O—H⋯N_pyridine_ hydrogen bonds with the *D* and *C* NIC cations, respectively (Table 2 ▶) (see Fig. 2 ▶). These cation–anion subunits are then extended into independent chain structures through pyrrolidinium N—H⋯O_sulfonate_ hydrogen bonds, which with anion *C* is three-centre (O51*B* ^i^ and O53*B* ^i^) and with anion *D*, linear (O52*A* ^ii^). These give independent zigzag chain substructures which extend along *b*. Although there are no formal hydrogen-bonding links between the two chains, there are π–π inter­actions between 5-SSA anion *A* and *B* benzene rings and *C* and *D* NIC cation pyridine rings, respectively [ring-centroid separations = 3.6422 (19) and 3.7117 (19) Å] (Fig. 4 ▶). The presence of a number of inter­molecular C—H⋯O hydrogen-bonding inter­actions to carboxyl, nitro and phenolic O-atom acceptors gives rise to an overall three-dimensional structure.

## Synthesis and crystallization   

The title salts (I) and (II) were prepared by refluxing equimolar qu­anti­ties of nicotine (160 mg) and the respective acids, 3,5-di­nitro­salicylic acid (230 mg) for (I) or 3-carb­oxy-4-hy­droxy­benzene­sulfonic acid (220 mg) for (II) in 30 ml of ethanol for 10 min, after which room temperature evaporation of the solutions gave, for (I), thin yellow needles and for (II) colourless prisms, from which specimens were cleaved for the X-ray analyses.

## Refinement details   

Crystal data, data collection and structure refinement details are summarized in Table 3 ▶. H atoms on all potentially inter­active O—H and N—H groups in all mol­ecular species, were located by difference-Fourier methods but these and the carbon-bound H-atoms were subsequently set as riding on the parent atoms in the refinement in calculated positions [O—H = 0.88, N—H = 0.94, C—H = 0.95–1.00 Å] and with *U*_iso_(H) = 1.5*U*_eq_(O or methyl-C) or 1.2*U*_eq_(C, N).

The site occupancy factors for the rotationally disordered phenolate components (O2*B*) and its other component (O21*B*) in anion *B* of (I) were determined as 0.752 (4): 0.248 (4) and were subsequently set at 0.75:0.25 in the refinement. 

In both structures, the known C21(*S*) absolute configuration was invoked. The Flack parameter for (I) [0.2 (16)] has no physical meaning. The absolute structure of compound (II) was confirmed by resonant scattering [Flack parameter = −0.02 (9)].

## Supplementary Material

Crystal structure: contains datablock(s) global, I, II. DOI: 10.1107/S1600536814023253/lh5736sup1.cif

Structure factors: contains datablock(s) I. DOI: 10.1107/S1600536814023253/lh5736Isup2.hkl

Structure factors: contains datablock(s) II. DOI: 10.1107/S1600536814023253/lh5736IIsup3.hkl

Supporting information file. DOI: 10.1107/S1600536814023253/lh5736Isup4.cml

Supporting information file. DOI: 10.1107/S1600536814023253/lh5736IIsup5.cml

CCDC references: 1030394, 1030395

Additional supporting information:  crystallographic information; 3D view; checkCIF report

## Figures and Tables

**Table 1:** Hydrogen-bond geometry (, ) for (I)

*D*H*A*	*D*H	H*A*	*D* *A*	*D*H*A*
O12*A*H12*A*O2*A*	0.84	1.71	2.475(4)	150
O12*B*H12*B*O2*B*	0.84	1.63	2.411(4)	152
N11*C*H11*C*N1*D* ^i^	0.93	1.89	2.809(4)	169
N11*D*H11*D*N1*C*	0.93	1.90	2.817(5)	168
C2*C*H2*C*O11*A* ^ii^	0.95	2.42	3.228(5)	143
C4*C*H4*C*O31*A* ^i^	0.95	2.59	3.452(5)	151
C6*C*H6*C*O32*A* ^iii^	0.95	2.27	3.054(5)	139
C11*C*H13*C*O32*B* ^i^	0.98	2.48	3.151(6)	126
C11*D*H14*D*O51*A* ^iv^	0.98	2.55	3.373(6)	141
C21*C*H21*C*O2*A* ^i^	1.00	2.27	3.163(5)	148
C21*D*H21*D*O11*B* ^v^	1.00	2.44	3.307(5)	144
C51*C*H52*C*O11*A* ^ii^	0.99	2.54	3.534(6)	177

Symmetry codes: (i) 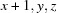; (ii) 
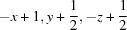
; (iii) 
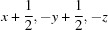
; (iv) 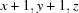; (v) 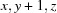.

**Table 2:** Hydrogen-bond geometry (, ) for (II)

*D*H*A*	*D*H	H*A*	*D* *A*	*D*H*A*
O2*A*H2*A*O12*A*	0.84	1.80	2.549(4)	147
O2*B*H2*B*O12*B*	0.84	1.82	2.561(4)	146
O11*A*H11*A*N1*D*	0.95	1.60	2.555(4)	179
O11*B*H11*B*N1*C*	0.95	1.61	2.558(4)	179
N11*C*H11*C*O51*B* ^i^	0.93	2.32	3.022(5)	132
N11*C*H11*C*O53*B* ^i^	0.93	2.15	3.029(5)	157
N11*D*H11*D*O52*A* ^ii^	0.93	1.85	2.735(4)	158
C11*D*H12*D*O2*B* ^iii^	0.98	2.51	3.491(5)	174
C2*C*H2*C*O53*B* ^i^	0.95	2.29	3.201(5)	160
C2*D*H2*D*O53*A* ^iv^	0.95	2.45	3.359(4)	160
C11*C*H12*C*O2*A* ^v^	0.98	2.52	3.481(5)	165
C11*C*H13*C*O52*B* ^vi^	0.98	2.46	3.290(5)	142
C11*D*H13*D*O51*A* ^iv^	0.98	2.37	3.251(5)	150
C21*C*H21*C*O52*B* ^vi^	1.00	2.42	3.331(5)	151

Symmetry codes: (i) 
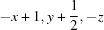
; (ii) 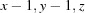; (iii) 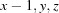; (iv) 
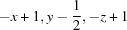
; (v) 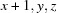; (vi) 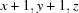.

**Table 3:** Experimental details

	(I)	(II)
Crystal data		
Chemical formula	C_10_H_15_N_2_^+^C_7_H_3_N_2_O_7_	C_10_H_15_N_2_^+^C_7_H_5_O_6_S
*M*_r_	390.35	380.41
Crystal system, space group	Orthorhombic, *P*2_1_2_1_2_1_	Monoclinic, *P*2_1_
Temperature (K)	200	200
*a*, *b*, *c* ()	6.8096(5), 17.6403(15), 29.3604(19)	7.1568(3), 12.6416(5), 19.1519(8)
, , ()	90, 90, 90	90, 93.729(4), 90
*V* (^3^)	3526.9(4)	1729.07(12)
*Z*	8	4
Radiation type	Mo *K*	Mo *K*
(mm^1^)	0.12	0.23
Crystal size (mm)	0.40 0.10 0.08	0.35 0.30 0.12

Data collection		
Diffractometer	Oxford Diffraction Gemini-S CCD detector	Oxford Diffraction Gemini-S CCD detector
Absorption correction	Multi-scan (*CrysAlis PRO*; Agilent, 2013 ▶)	Multi-scan (*CrysAlis PRO*; Agilent, 2013 ▶)
*T*_min_, *T*_max_	0.807, 0.980	0.909, 0.981
No. of measured, independent and observed [*I* > 2(*I*)] reflections	8840, 6476, 4303	7764, 5104, 4424
*R*_int_	0.028	0.031
(sin /)_max_ (^1^)	0.617	0.680

Refinement		
*R*[*F* ^2^ > 2(*F* ^2^)], *wR*(*F* ^2^), *S*	0.072, 0.122, 1.07	0.046, 0.108, 1.01
No. of reflections	6476	5104
No. of parameters	508	469
No. of restraints	2	1
H-atom treatment	H-atom parameters constrained	H-atom parameters constrained
_max_, _min_ (e ^3^)	0.44, 0.19	0.49, 0.36
Absolute structure	Flack (1983 ▶), 2983 Friedel pairs	Flack (1983 ▶), 4361 Friedel pairs
Absolute structure parameter	0.2(16)	0.02(9)

Computer programs: *CrysAlis PRO* (Agilent, 2013 ▶), *SHELXS97* and *SHELXL97* (Sheldrick, 2008 ▶) within *WinGX* (Farrugia, 2012 ▶), *SIR92* (Altomare *et al.*, 1993 ▶)and *PLATON* (Spek, 2009 ▶).

## supplementary crystallographic information

### Crystal data

**Table:**

C_10_H_15_N_2_^+^·C_7_H_5_O_6_S^−^	*F*(000) = 800
*M**_r_* = 380.41	*D*_x_ = 1.461 Mg m^−^^3^
Monoclinic, *P*2_1_	Mo *K*α radiation, λ = 0.71073 Å
Hall symbol: P 2yb	Cell parameters from 2239 reflections
*a* = 7.1568 (3) Å	θ = 3.4–27.5°
*b* = 12.6416 (5) Å	µ = 0.23 mm^−^^1^
*c* = 19.1519 (8) Å	*T* = 200 K
β = 93.729 (4)°	Prism, colourless
*V* = 1729.07 (12) Å^3^	0.35 × 0.30 × 0.12 mm
*Z* = 4	

### Data collection

**Table:**

Oxford Diffraction Gemini-S CCD-detector diffractometer	5104 independent reflections
Radiation source: Enhance (Mo) X-ray source	4424 reflections with *I* > 2σ(*I*)
Graphite monochromator	*R*_int_ = 0.031
Detector resolution: 16.077 pixels mm^-1^	θ_max_ = 28.9°, θ_min_ = 3.2°
ω scans	*h* = −8→9
Absorption correction: multi-scan (*CrysAlis PRO*; Agilent, 2013)	*k* = −17→8
*T*_min_ = 0.909, *T*_max_ = 0.981	*l* = −24→14
7764 measured reflections	

### Refinement

**Table:**

Refinement on *F*^2^	Secondary atom site location: difference Fourier map
Least-squares matrix: full	Hydrogen site location: inferred from neighbouring sites
*R*[*F*^2^ > 2σ(*F*^2^)] = 0.046	H-atom parameters constrained
*wR*(*F*^2^) = 0.108	*w* = 1/[σ^2^(*F*_o_^2^) + (0.0448*P*)^2^ + 0.6152*P*] where *P* = (*F*_o_^2^ + 2*F*_c_^2^)/3
*S* = 1.01	(Δ/σ)_max_ < 0.001
5104 reflections	Δρ_max_ = 0.49 e Å^−^^3^
469 parameters	Δρ_min_ = −0.36 e Å^−^^3^
1 restraint	Absolute structure: Flack (1983), 4361 Friedel pairs
Primary atom site location: structure-invariant direct methods	Absolute structure parameter: −0.02 (9)

### Special details

**Table:**

Geometry. Bond distances, angles *etc*. have been calculated using the rounded fractional coordinates. All su's are estimated from the variances of the (full) variance-covariance matrix. The cell e.s.d.'s are taken into account in the estimation of distances, angles and torsion angles
Refinement. Refinement of *F*^2^ against ALL reflections. The weighted *R*-factor *wR* and goodness of fit *S* are based on *F*^2^, conventional *R*-factors *R* are based on *F*, with *F* set to zero for negative *F*^2^. The threshold expression of *F*^2^ > σ(*F*^2^) is used only for calculating *R*-factors(gt) *etc*. and is not relevant to the choice of reflections for refinement. *R*-factors based on *F*^2^ are statistically about twice as large as those based on *F*, and *R*- factors based on ALL data will be even larger.

### Fractional atomic coordinates and isotropic or equivalent isotropic displacement parameters (Å^2^)

**Table:**

	*x*	*y*	*z*	*U*_iso_*/*U*_eq_	
S5A	0.68704 (12)	0.55842 (7)	0.41012 (4)	0.0263 (3)	
O2A	0.3397 (4)	0.3912 (2)	0.14516 (12)	0.0340 (8)	
O11A	0.3204 (4)	0.2139 (2)	0.32641 (13)	0.0348 (8)	
O12A	0.2584 (4)	0.2236 (2)	0.21069 (13)	0.0337 (8)	
O51A	0.8728 (4)	0.5131 (3)	0.41339 (15)	0.0508 (10)	
O52A	0.6922 (4)	0.6735 (2)	0.40180 (14)	0.0422 (9)	
O53A	0.5792 (4)	0.5253 (2)	0.46723 (12)	0.0393 (9)	
C1A	0.4129 (4)	0.3676 (3)	0.26886 (17)	0.0221 (9)	
C2A	0.4124 (5)	0.4284 (3)	0.20674 (17)	0.0222 (10)	
C3A	0.4845 (5)	0.5304 (3)	0.20917 (17)	0.0240 (10)	
C4A	0.5643 (4)	0.5705 (3)	0.27120 (17)	0.0239 (9)	
C5A	0.5696 (4)	0.5108 (3)	0.33217 (17)	0.0211 (9)	
C6A	0.4930 (5)	0.4106 (3)	0.33044 (18)	0.0234 (10)	
C11A	0.3243 (5)	0.2614 (3)	0.26686 (19)	0.0259 (11)	
S5B	0.30149 (14)	−0.14181 (8)	0.08257 (5)	0.0345 (3)	
O2B	0.6834 (4)	0.0076 (2)	0.34578 (12)	0.0344 (8)	
O11B	0.7029 (4)	0.1928 (2)	0.16767 (13)	0.0347 (8)	
O12B	0.7680 (4)	0.1780 (2)	0.28273 (13)	0.0335 (8)	
O51B	0.1062 (4)	−0.1205 (3)	0.09470 (17)	0.0625 (11)	
O52B	0.3370 (4)	−0.2529 (2)	0.07618 (16)	0.0480 (10)	
O53B	0.3571 (6)	−0.0759 (3)	0.02651 (16)	0.0801 (15)	
C1B	0.6053 (4)	0.0375 (3)	0.22345 (17)	0.0206 (9)	
C2B	0.6058 (5)	−0.0256 (3)	0.28333 (17)	0.0233 (10)	
C3B	0.5299 (5)	−0.1274 (3)	0.28007 (18)	0.0255 (10)	
C4B	0.4433 (5)	−0.1631 (3)	0.21848 (19)	0.0261 (10)	
C5B	0.4331 (5)	−0.0982 (3)	0.15869 (17)	0.0231 (10)	
C6B	0.5145 (4)	0.0004 (3)	0.16130 (17)	0.0222 (10)	
C11B	0.6976 (5)	0.1427 (3)	0.22658 (19)	0.0260 (11)	
N1C	0.8536 (4)	0.3758 (3)	0.18074 (15)	0.0270 (9)	
N11C	0.9801 (4)	0.5668 (3)	−0.00002 (14)	0.0295 (9)	
C2C	0.8558 (5)	0.4408 (3)	0.12534 (19)	0.0253 (10)	
C3C	0.9371 (4)	0.5393 (3)	0.12903 (18)	0.0257 (10)	
C4C	1.0161 (5)	0.5724 (3)	0.19371 (18)	0.0294 (10)	
C5C	1.0096 (5)	0.5074 (3)	0.25102 (18)	0.0296 (11)	
C6C	0.9288 (5)	0.4092 (3)	0.24258 (19)	0.0297 (11)	
C11C	1.1769 (5)	0.5314 (4)	−0.0030 (2)	0.0392 (14)	
C21C	0.9336 (5)	0.6148 (3)	0.06816 (18)	0.0290 (11)	
C31C	0.7439 (6)	0.6662 (4)	0.0489 (2)	0.0493 (16)	
C41C	0.7621 (7)	0.7097 (4)	−0.0242 (2)	0.0529 (17)	
C51C	0.9232 (6)	0.6511 (4)	−0.0534 (2)	0.0476 (16)	
N1D	0.1735 (4)	0.0297 (2)	0.31881 (15)	0.0257 (8)	
N11D	−0.0524 (4)	−0.2131 (3)	0.48368 (14)	0.0327 (10)	
C2D	0.1839 (5)	−0.0236 (3)	0.37895 (18)	0.0239 (10)	
C3D	0.1071 (4)	−0.1235 (3)	0.38558 (16)	0.0211 (9)	
C4D	0.0196 (5)	−0.1695 (3)	0.32573 (17)	0.0256 (10)	
C5D	0.0119 (5)	−0.1153 (3)	0.26338 (18)	0.0283 (11)	
C6D	0.0898 (5)	−0.0152 (3)	0.26175 (19)	0.0284 (11)	
C11D	−0.1872 (6)	−0.1290 (4)	0.4998 (2)	0.0430 (14)	
C21D	0.1287 (5)	−0.1769 (3)	0.45539 (17)	0.0267 (10)	
C31D	0.2434 (6)	−0.2778 (4)	0.4595 (2)	0.0442 (14)	
C41D	0.1992 (8)	−0.3244 (4)	0.5301 (2)	0.0626 (19)	
C51D	0.0103 (7)	−0.2808 (4)	0.5453 (2)	0.0564 (16)	
H2A	0.30050	0.32920	0.15050	0.0510*	
H3A	0.47890	0.57260	0.16810	0.0290*	
H4A	0.61610	0.63970	0.27220	0.0290*	
H6A	0.49510	0.37020	0.37230	0.0280*	
H11A	0.26610	0.14530	0.32280	0.0520*	
H2B	0.71920	0.07050	0.34230	0.0520*	
H3B	0.53810	−0.17170	0.32020	0.0310*	
H4B	0.39000	−0.23190	0.21620	0.0310*	
H6B	0.50900	0.04360	0.12060	0.0270*	
H11B	0.75890	0.26070	0.17310	0.0520*	
H2C	0.79870	0.41780	0.08180	0.0300*	
H4C	1.07430	0.63980	0.19820	0.0350*	
H5A	1.06000	0.53000	0.29570	0.0350*	
H6C	0.92630	0.36360	0.28190	0.0360*	
H11C	0.90280	0.50850	−0.00850	0.0350*	
H12C	1.20770	0.48160	0.03520	0.0580*	
H13C	1.26050	0.59270	0.00170	0.0580*	
H21C	1.02610	0.67240	0.07990	0.0350*	
H31C	0.64180	0.61330	0.04910	0.0590*	
H32C	0.71820	0.72360	0.08200	0.0590*	
H41C	0.64530	0.69750	−0.05370	0.0630*	
H42C	0.78790	0.78660	−0.02240	0.0630*	
H51C	1.02880	0.69990	−0.06010	0.0570*	
H52C	0.88390	0.61850	−0.09900	0.0570*	
H134	1.19250	0.49640	−0.04800	0.0580*	
H12D	−0.21690	−0.08640	0.45780	0.0650*	
H2D	0.24650	0.00810	0.41890	0.0290*	
H4D	−0.03450	−0.23790	0.32810	0.0310*	
H5D	−0.04590	−0.14600	0.22220	0.0340*	
H6D	0.08380	0.02270	0.21880	0.0340*	
H11D	−0.11170	−0.25710	0.45020	0.0390*	
H21D	0.18880	−0.12540	0.48950	0.0320*	
H13D	−0.13180	−0.08360	0.53710	0.0650*	
H14D	−0.30210	−0.16130	0.51510	0.0650*	
H31D	0.37870	−0.26240	0.45810	0.0530*	
H32D	0.20480	−0.32650	0.42080	0.0530*	
H41D	0.19550	−0.40260	0.52770	0.0750*	
H42D	0.29520	−0.30300	0.56690	0.0750*	
H51D	−0.07990	−0.33910	0.55110	0.0680*	
H52D	0.01960	−0.23820	0.58870	0.0680*	

### Atomic displacement parameters (Å^2^)

**Table:**

	*U*^11^	*U*^22^	*U*^33^	*U*^12^	*U*^13^	*U*^23^
S5A	0.0295 (4)	0.0253 (5)	0.0232 (4)	−0.0031 (4)	−0.0062 (3)	0.0026 (4)
O2A	0.0467 (15)	0.0280 (15)	0.0261 (13)	−0.0022 (13)	−0.0058 (11)	−0.0012 (11)
O11A	0.0456 (15)	0.0234 (14)	0.0352 (14)	−0.0115 (13)	0.0018 (11)	0.0042 (12)
O12A	0.0397 (14)	0.0249 (14)	0.0360 (14)	−0.0065 (13)	−0.0013 (11)	−0.0045 (12)
O51A	0.0347 (14)	0.073 (2)	0.0424 (16)	0.0124 (17)	−0.0146 (12)	0.0011 (17)
O52A	0.0580 (18)	0.0242 (14)	0.0417 (16)	−0.0116 (14)	−0.0180 (13)	0.0052 (13)
O53A	0.0496 (15)	0.0460 (17)	0.0219 (12)	−0.0115 (15)	−0.0002 (10)	0.0003 (12)
C1A	0.0201 (16)	0.0212 (17)	0.0249 (16)	0.0028 (16)	0.0010 (12)	0.0024 (15)
C2A	0.0238 (17)	0.0227 (18)	0.0197 (16)	0.0035 (15)	−0.0009 (13)	0.0010 (14)
C3A	0.0271 (17)	0.0256 (18)	0.0193 (16)	0.0050 (16)	0.0008 (13)	0.0056 (14)
C4A	0.0213 (15)	0.0197 (17)	0.0305 (17)	−0.0002 (16)	0.0009 (13)	0.0055 (16)
C5A	0.0221 (16)	0.0196 (17)	0.0210 (16)	−0.0011 (16)	−0.0025 (12)	0.0008 (14)
C6A	0.0264 (18)	0.0203 (18)	0.0233 (17)	0.0010 (16)	0.0002 (13)	0.0055 (14)
C11A	0.0231 (17)	0.0221 (19)	0.0326 (19)	0.0001 (15)	0.0022 (14)	−0.0005 (16)
S5B	0.0413 (5)	0.0300 (5)	0.0305 (5)	−0.0046 (5)	−0.0110 (4)	−0.0026 (4)
O2B	0.0499 (15)	0.0292 (14)	0.0227 (12)	−0.0051 (14)	−0.0078 (11)	−0.0009 (11)
O11B	0.0440 (15)	0.0269 (15)	0.0330 (14)	−0.0111 (13)	0.0007 (11)	0.0065 (12)
O12B	0.0442 (15)	0.0270 (14)	0.0286 (14)	−0.0052 (13)	−0.0029 (11)	−0.0020 (12)
O51B	0.0379 (16)	0.070 (2)	0.075 (2)	0.0237 (18)	−0.0305 (15)	−0.029 (2)
O52B	0.0402 (16)	0.0333 (17)	0.068 (2)	0.0016 (14)	−0.0145 (14)	−0.0229 (16)
O53B	0.126 (3)	0.084 (3)	0.0272 (16)	−0.057 (3)	−0.0197 (18)	0.0087 (18)
C1B	0.0202 (15)	0.0139 (17)	0.0277 (16)	0.0026 (14)	0.0020 (12)	−0.0005 (13)
C2B	0.0234 (17)	0.0248 (19)	0.0218 (17)	0.0048 (16)	0.0013 (13)	−0.0008 (15)
C3B	0.0310 (18)	0.0187 (17)	0.0263 (17)	0.0035 (17)	−0.0021 (13)	0.0067 (15)
C4B	0.0242 (17)	0.0175 (18)	0.0358 (19)	0.0016 (15)	−0.0052 (14)	0.0029 (15)
C5B	0.0211 (16)	0.0219 (18)	0.0260 (17)	0.0044 (15)	−0.0002 (13)	0.0007 (15)
C6B	0.0222 (16)	0.0218 (18)	0.0224 (16)	0.0028 (15)	−0.0001 (13)	0.0025 (14)
C11B	0.0249 (17)	0.0193 (18)	0.034 (2)	0.0029 (15)	0.0047 (14)	0.0001 (16)
N1C	0.0256 (14)	0.0258 (17)	0.0294 (15)	0.0004 (14)	0.0003 (11)	0.0076 (14)
N11C	0.0363 (15)	0.0269 (16)	0.0251 (14)	−0.0033 (15)	0.0003 (11)	0.0064 (14)
C2C	0.0250 (18)	0.0242 (19)	0.0263 (17)	0.0007 (16)	−0.0012 (13)	0.0008 (15)
C3C	0.0225 (16)	0.0238 (19)	0.0312 (18)	0.0015 (15)	0.0041 (13)	0.0038 (15)
C4C	0.0239 (16)	0.0269 (19)	0.0375 (19)	−0.0031 (16)	0.0036 (14)	−0.0039 (17)
C5C	0.0255 (18)	0.038 (2)	0.0249 (18)	0.0011 (17)	−0.0003 (14)	−0.0009 (17)
C6C	0.0263 (18)	0.035 (2)	0.0278 (18)	0.0032 (17)	0.0025 (14)	0.0108 (17)
C11C	0.038 (2)	0.044 (3)	0.036 (2)	−0.003 (2)	0.0049 (16)	−0.006 (2)
C21C	0.0339 (19)	0.0202 (17)	0.0333 (19)	0.0005 (16)	0.0064 (14)	0.0042 (16)
C31C	0.048 (2)	0.043 (3)	0.058 (3)	0.020 (2)	0.013 (2)	0.024 (2)
C41C	0.064 (3)	0.038 (3)	0.055 (3)	0.005 (2)	−0.009 (2)	0.020 (2)
C51C	0.060 (3)	0.048 (3)	0.035 (2)	0.003 (2)	0.0038 (19)	0.024 (2)
N1D	0.0265 (14)	0.0213 (15)	0.0299 (15)	−0.0009 (13)	0.0067 (11)	0.0047 (13)
N11D	0.0428 (18)	0.0322 (17)	0.0230 (15)	−0.0165 (15)	0.0009 (12)	−0.0011 (14)
C2D	0.0264 (17)	0.0190 (17)	0.0265 (17)	0.0002 (15)	0.0033 (13)	0.0007 (14)
C3D	0.0215 (15)	0.0182 (17)	0.0239 (16)	0.0011 (15)	0.0033 (12)	−0.0009 (14)
C4D	0.0266 (17)	0.0226 (18)	0.0278 (18)	−0.0038 (15)	0.0038 (13)	0.0005 (15)
C5D	0.0253 (18)	0.035 (2)	0.0241 (17)	−0.0020 (17)	−0.0020 (13)	0.0008 (16)
C6D	0.0281 (18)	0.033 (2)	0.0245 (18)	0.0016 (17)	0.0058 (14)	0.0052 (16)
C11D	0.040 (2)	0.052 (3)	0.038 (2)	−0.010 (2)	0.0113 (17)	−0.012 (2)
C21D	0.0310 (18)	0.0252 (18)	0.0231 (17)	−0.0095 (16)	−0.0031 (13)	−0.0007 (14)
C31D	0.058 (3)	0.037 (2)	0.036 (2)	0.012 (2)	−0.0081 (19)	0.005 (2)
C41D	0.099 (4)	0.039 (3)	0.048 (3)	0.011 (3)	−0.008 (3)	0.019 (2)
C51D	0.077 (3)	0.061 (3)	0.030 (2)	−0.022 (3)	−0.006 (2)	0.023 (2)

### Geometric parameters (Å, º)

**Table:**

S5A—O51A	1.445 (3)	C3B—H3B	0.9500
S5A—O52A	1.464 (3)	C4B—H4B	0.9500
S5A—O53A	1.441 (3)	C6B—H6B	0.9500
S5A—C5A	1.770 (3)	C2C—C3C	1.374 (5)
S5B—O51B	1.457 (3)	C3C—C21C	1.506 (5)
S5B—O52B	1.434 (3)	C3C—C4C	1.392 (5)
S5B—O53B	1.436 (4)	C4C—C5C	1.374 (5)
S5B—C5B	1.771 (4)	C5C—C6C	1.375 (5)
O2A—C2A	1.343 (4)	C21C—C31C	1.529 (6)
O11A—C11A	1.291 (4)	C31C—C41C	1.518 (6)
O12A—C11A	1.242 (4)	C41C—C51C	1.508 (7)
O2A—H2A	0.8400	C2C—H2C	0.9500
O11A—H11A	0.9500	C4C—H4C	0.9500
O2B—C2B	1.352 (4)	C5C—H5A	0.9500
O11B—C11B	1.297 (4)	C6C—H6C	0.9500
O12B—C11B	1.241 (4)	C11C—H134	0.9800
O2B—H2B	0.8400	C11C—H12C	0.9800
O11B—H11B	0.9500	C11C—H13C	0.9800
N1C—C6C	1.337 (5)	C21C—H21C	1.0000
N1C—C2C	1.343 (5)	C31C—H32C	0.9900
N11C—C11C	1.483 (5)	C31C—H31C	0.9900
N11C—C51C	1.514 (6)	C41C—H41C	0.9900
N11C—C21C	1.497 (5)	C41C—H42C	0.9900
N11C—H11C	0.9300	C51C—H52C	0.9900
N1D—C2D	1.332 (4)	C51C—H51C	0.9900
N1D—C6D	1.338 (5)	C2D—C3D	1.386 (5)
N11D—C51D	1.503 (5)	C3D—C21D	1.497 (5)
N11D—C11D	1.482 (6)	C3D—C4D	1.397 (5)
N11D—C21D	1.508 (5)	C4D—C5D	1.375 (5)
N11D—H11D	0.9300	C5D—C6D	1.384 (5)
C1A—C2A	1.416 (5)	C21D—C31D	1.516 (6)
C1A—C11A	1.484 (5)	C31D—C41D	1.527 (6)
C1A—C6A	1.388 (5)	C41D—C51D	1.506 (7)
C2A—C3A	1.389 (5)	C2D—H2D	0.9500
C3A—C4A	1.381 (5)	C4D—H4D	0.9500
C4A—C5A	1.389 (5)	C5D—H5D	0.9500
C5A—C6A	1.380 (5)	C6D—H6D	0.9500
C3A—H3A	0.9500	C11D—H12D	0.9800
C4A—H4A	0.9500	C11D—H13D	0.9800
C6A—H6A	0.9500	C11D—H14D	0.9800
C1B—C6B	1.399 (5)	C21D—H21D	1.0000
C1B—C11B	1.484 (5)	C31D—H31D	0.9900
C1B—C2B	1.397 (5)	C31D—H32D	0.9900
C2B—C3B	1.397 (5)	C41D—H41D	0.9900
C3B—C4B	1.373 (5)	C41D—H42D	0.9900
C4B—C5B	1.407 (5)	C51D—H51D	0.9900
C5B—C6B	1.375 (5)	C51D—H52D	0.9900
			
O51A—S5A—O52A	111.65 (19)	N11C—C21C—C31C	101.9 (3)
O51A—S5A—O53A	112.86 (17)	C21C—C31C—C41C	104.4 (3)
O51A—S5A—C5A	106.66 (17)	C31C—C41C—C51C	106.1 (4)
O52A—S5A—O53A	112.94 (16)	N11C—C51C—C41C	105.9 (3)
O52A—S5A—C5A	105.07 (17)	N1C—C2C—H2C	119.00
O53A—S5A—C5A	107.03 (15)	C3C—C2C—H2C	119.00
O51B—S5B—C5B	105.99 (18)	C5C—C4C—H4C	120.00
O51B—S5B—O52B	111.8 (2)	C3C—C4C—H4C	120.00
O51B—S5B—O53B	109.1 (2)	C4C—C5C—H5A	121.00
O53B—S5B—C5B	106.1 (2)	C6C—C5C—H5A	121.00
O52B—S5B—O53B	116.4 (2)	C5C—C6C—H6C	119.00
O52B—S5B—C5B	106.70 (18)	N1C—C6C—H6C	119.00
C2A—O2A—H2A	109.00	N11C—C11C—H13C	109.00
C11A—O11A—H11A	113.00	H12C—C11C—H13C	109.00
C2B—O2B—H2B	109.00	N11C—C11C—H12C	109.00
C11B—O11B—H11B	112.00	H13C—C11C—H134	109.00
C2C—N1C—C6C	118.8 (4)	N11C—C11C—H134	109.00
C11C—N11C—C21C	114.9 (3)	H12C—C11C—H134	109.00
C11C—N11C—C51C	113.6 (3)	C31C—C21C—H21C	108.00
C21C—N11C—C51C	103.8 (3)	C3C—C21C—H21C	108.00
C51C—N11C—H11C	108.00	N11C—C21C—H21C	108.00
C21C—N11C—H11C	108.00	C41C—C31C—H32C	111.00
C11C—N11C—H11C	108.00	C21C—C31C—H31C	111.00
C2D—N1D—C6D	119.2 (3)	C41C—C31C—H31C	111.00
C11D—N11D—C51D	114.2 (3)	H31C—C31C—H32C	109.00
C11D—N11D—C21D	116.3 (3)	C21C—C31C—H32C	111.00
C21D—N11D—C51D	103.6 (3)	C51C—C41C—H41C	110.00
C51D—N11D—H11D	107.00	H41C—C41C—H42C	109.00
C11D—N11D—H11D	107.00	C31C—C41C—H42C	111.00
C21D—N11D—H11D	107.00	C31C—C41C—H41C	110.00
C6A—C1A—C11A	121.9 (3)	C51C—C41C—H42C	110.00
C2A—C1A—C11A	119.4 (3)	H51C—C51C—H52C	109.00
C2A—C1A—C6A	118.7 (3)	N11C—C51C—H51C	111.00
O2A—C2A—C1A	121.8 (3)	C41C—C51C—H52C	111.00
O2A—C2A—C3A	118.5 (3)	C41C—C51C—H51C	111.00
C1A—C2A—C3A	119.7 (3)	N11C—C51C—H52C	111.00
C2A—C3A—C4A	120.1 (3)	N1D—C2D—C3D	122.7 (3)
C3A—C4A—C5A	120.7 (3)	C2D—C3D—C21D	118.3 (3)
C4A—C5A—C6A	119.3 (3)	C2D—C3D—C4D	117.6 (3)
S5A—C5A—C4A	120.7 (3)	C4D—C3D—C21D	124.1 (3)
S5A—C5A—C6A	119.9 (3)	C3D—C4D—C5D	119.7 (3)
C1A—C6A—C5A	121.4 (3)	C4D—C5D—C6D	118.8 (3)
O11A—C11A—C1A	115.6 (3)	N1D—C6D—C5D	121.9 (3)
O12A—C11A—C1A	120.5 (3)	C3D—C21D—C31D	116.7 (3)
O11A—C11A—O12A	123.9 (3)	N11D—C21D—C3D	114.6 (3)
C2A—C3A—H3A	120.00	N11D—C21D—C31D	101.6 (3)
C4A—C3A—H3A	120.00	C21D—C31D—C41D	103.2 (3)
C3A—C4A—H4A	120.00	C31D—C41D—C51D	105.7 (4)
C5A—C4A—H4A	120.00	N11D—C51D—C41D	106.4 (3)
C5A—C6A—H6A	119.00	N1D—C2D—H2D	119.00
C1A—C6A—H6A	119.00	C3D—C2D—H2D	119.00
C6B—C1B—C11B	121.0 (3)	C3D—C4D—H4D	120.00
C2B—C1B—C11B	120.1 (3)	C5D—C4D—H4D	120.00
C2B—C1B—C6B	118.9 (3)	C4D—C5D—H5D	121.00
C1B—C2B—C3B	120.6 (3)	C6D—C5D—H5D	121.00
O2B—C2B—C3B	117.5 (3)	N1D—C6D—H6D	119.00
O2B—C2B—C1B	121.9 (3)	C5D—C6D—H6D	119.00
C2B—C3B—C4B	119.6 (3)	N11D—C11D—H12D	109.00
C3B—C4B—C5B	120.3 (3)	N11D—C11D—H13D	110.00
C4B—C5B—C6B	120.0 (3)	N11D—C11D—H14D	109.00
S5B—C5B—C4B	119.0 (3)	H12D—C11D—H13D	109.00
S5B—C5B—C6B	120.9 (3)	H12D—C11D—H14D	109.00
C1B—C6B—C5B	120.5 (3)	H13D—C11D—H14D	109.00
O12B—C11B—C1B	120.8 (3)	N11D—C21D—H21D	108.00
O11B—C11B—C1B	116.1 (3)	C3D—C21D—H21D	108.00
O11B—C11B—O12B	123.1 (3)	C31D—C21D—H21D	108.00
C2B—C3B—H3B	120.00	C21D—C31D—H31D	111.00
C4B—C3B—H3B	120.00	C21D—C31D—H32D	111.00
C3B—C4B—H4B	120.00	C41D—C31D—H31D	111.00
C5B—C4B—H4B	120.00	C41D—C31D—H32D	111.00
C5B—C6B—H6B	120.00	H31D—C31D—H32D	109.00
C1B—C6B—H6B	120.00	C31D—C41D—H41D	111.00
N1C—C2C—C3C	122.7 (3)	C31D—C41D—H42D	111.00
C2C—C3C—C4C	117.7 (3)	C51D—C41D—H41D	111.00
C4C—C3C—C21C	118.9 (3)	C51D—C41D—H42D	111.00
C2C—C3C—C21C	123.3 (3)	H41D—C41D—H42D	109.00
C3C—C4C—C5C	119.9 (3)	N11D—C51D—H51D	110.00
C4C—C5C—C6C	118.8 (3)	N11D—C51D—H52D	111.00
N1C—C6C—C5C	122.1 (3)	C41D—C51D—H51D	110.00
N11C—C21C—C3C	115.2 (3)	C41D—C51D—H52D	110.00
C3C—C21C—C31C	115.2 (3)	H51D—C51D—H52D	109.00
			
O51A—S5A—C5A—C4A	−95.6 (3)	C6B—C1B—C2B—O2B	−176.9 (3)
O51A—S5A—C5A—C6A	80.1 (3)	C6B—C1B—C2B—C3B	5.0 (5)
O52A—S5A—C5A—C4A	23.0 (3)	C11B—C1B—C2B—C3B	−175.9 (3)
O52A—S5A—C5A—C6A	−161.3 (3)	C2B—C1B—C6B—C5B	−2.3 (5)
O53A—S5A—C5A—C4A	143.4 (3)	C11B—C1B—C6B—C5B	178.6 (3)
O53A—S5A—C5A—C6A	−41.0 (3)	C2B—C1B—C11B—O11B	175.1 (3)
O53B—S5B—C5B—C6B	19.4 (4)	C2B—C1B—C11B—O12B	−3.5 (5)
O52B—S5B—C5B—C4B	−40.0 (3)	C6B—C1B—C11B—O11B	−5.8 (5)
O51B—S5B—C5B—C4B	79.3 (3)	C11B—C1B—C2B—O2B	2.2 (5)
O51B—S5B—C5B—C6B	−96.5 (3)	C6B—C1B—C11B—O12B	175.6 (3)
O52B—S5B—C5B—C6B	144.1 (3)	O2B—C2B—C3B—C4B	177.5 (3)
O53B—S5B—C5B—C4B	−164.8 (3)	C1B—C2B—C3B—C4B	−4.2 (5)
C2C—N1C—C6C—C5C	−0.6 (5)	C2B—C3B—C4B—C5B	0.8 (5)
C6C—N1C—C2C—C3C	1.9 (5)	C3B—C4B—C5B—C6B	1.7 (5)
C21C—N11C—C51C—C41C	−30.3 (4)	C3B—C4B—C5B—S5B	−174.1 (3)
C11C—N11C—C21C—C3C	−67.9 (4)	C4B—C5B—C6B—C1B	−1.0 (5)
C11C—N11C—C51C—C41C	−155.8 (4)	S5B—C5B—C6B—C1B	174.8 (2)
C51C—N11C—C21C—C31C	41.9 (4)	N1C—C2C—C3C—C4C	−1.4 (5)
C11C—N11C—C21C—C31C	166.6 (4)	N1C—C2C—C3C—C21C	−177.4 (3)
C51C—N11C—C21C—C3C	167.4 (3)	C2C—C3C—C4C—C5C	−0.5 (5)
C2D—N1D—C6D—C5D	0.6 (5)	C21C—C3C—C4C—C5C	175.7 (3)
C6D—N1D—C2D—C3D	−1.6 (5)	C2C—C3C—C21C—N11C	−45.7 (4)
C51D—N11D—C21D—C3D	169.5 (3)	C2C—C3C—C21C—C31C	72.5 (5)
C11D—N11D—C21D—C31D	168.9 (3)	C4C—C3C—C21C—C31C	−103.4 (4)
C21D—N11D—C51D—C41D	−27.3 (4)	C4C—C3C—C21C—N11C	138.4 (3)
C51D—N11D—C21D—C31D	42.6 (3)	C3C—C4C—C5C—C6C	1.7 (5)
C11D—N11D—C51D—C41D	−154.9 (4)	C4C—C5C—C6C—N1C	−1.2 (6)
C11D—N11D—C21D—C3D	−64.3 (4)	N11C—C21C—C31C—C41C	−38.1 (4)
C2A—C1A—C11A—O12A	3.6 (5)	C3C—C21C—C31C—C41C	−163.5 (3)
C2A—C1A—C11A—O11A	−175.9 (3)	C21C—C31C—C41C—C51C	19.6 (5)
C6A—C1A—C11A—O11A	2.6 (5)	C31C—C41C—C51C—N11C	6.2 (5)
C6A—C1A—C11A—O12A	−178.0 (3)	N1D—C2D—C3D—C4D	1.4 (5)
C6A—C1A—C2A—C3A	−2.6 (5)	N1D—C2D—C3D—C21D	179.3 (3)
C11A—C1A—C2A—O2A	−2.4 (5)	C2D—C3D—C4D—C5D	−0.2 (5)
C11A—C1A—C2A—C3A	176.0 (3)	C21D—C3D—C4D—C5D	−177.9 (3)
C2A—C1A—C6A—C5A	0.6 (5)	C2D—C3D—C21D—N11D	125.7 (3)
C11A—C1A—C6A—C5A	−177.9 (3)	C2D—C3D—C21D—C31D	−115.7 (4)
C6A—C1A—C2A—O2A	179.1 (3)	C4D—C3D—C21D—N11D	−56.6 (5)
C1A—C2A—C3A—C4A	3.1 (5)	C4D—C3D—C21D—C31D	62.0 (5)
O2A—C2A—C3A—C4A	−178.5 (3)	C3D—C4D—C5D—C6D	−0.8 (5)
C2A—C3A—C4A—C5A	−1.6 (5)	C4D—C5D—C6D—N1D	0.6 (5)
C3A—C4A—C5A—C6A	−0.4 (5)	N11D—C21D—C31D—C41D	−41.6 (4)
C3A—C4A—C5A—S5A	175.3 (3)	C3D—C21D—C31D—C41D	−167.0 (3)
C4A—C5A—C6A—C1A	0.9 (5)	C21D—C31D—C41D—C51D	25.0 (5)
S5A—C5A—C6A—C1A	−174.9 (3)	C31D—C41D—C51D—N11D	1.3 (5)

### Hydrogen-bond geometry (Å, º)

**Table:**

*D*—H···*A*	*D*—H	H···*A*	*D*···*A*	*D*—H···*A*
O2*A*—H2*A*···O12*A*	0.84	1.80	2.549 (4)	147
O2*B*—H2*B*···O12*B*	0.84	1.82	2.561 (4)	146
O11*A*—H11*A*···N1*D*	0.95	1.60	2.555 (4)	179
O11*B*—H11*B*···N1*C*	0.95	1.61	2.558 (4)	179
N11*C*—H11*C*···O51*B*^i^	0.93	2.32	3.022 (5)	132
N11*C*—H11*C*···O53*B*^i^	0.93	2.15	3.029 (5)	157
N11*D*—H11*D*···O52*A*^ii^	0.93	1.85	2.735 (4)	158
C11*D*—H12*D*···O2*B*^iii^	0.98	2.51	3.491 (5)	174
C2*C*—H2*C*···O53*B*^i^	0.95	2.29	3.201 (5)	160
C2*D*—H2*D*···O53*A*^iv^	0.95	2.45	3.359 (4)	160
C4*A*—H4*A*···O52*A*	0.95	2.54	2.914 (4)	103
C6*B*—H6*B*···O53*B*	0.95	2.54	2.913 (5)	103
C11*C*—H12*C*···O2*A*^v^	0.98	2.52	3.481 (5)	165
C11*C*—H13*C*···O52*B*^vi^	0.98	2.46	3.290 (5)	142
C11*D*—H13*D*···O51*A*^iv^	0.98	2.37	3.251 (5)	150
C21*C*—H21*C*···O52*B*^vi^	1.00	2.42	3.331 (5)	151

Symmetry codes: (i) −*x*+1, *y*+1/2, −*z*; (ii) *x*−1, *y*−1, *z*; (iii) *x*−1, *y*, *z*; (iv) −*x*+1, *y*−1/2, −*z*+1; (v) *x*+1, *y*, *z*; (vi) *x*+1, *y*+1, *z*.
